# The use of simple reparameterizations to improve the efficiency of Markov chain Monte Carlo estimation for multilevel models with applications to discrete time survival models

**DOI:** 10.1111/j.1467-985X.2009.00586.x

**Published:** 2009-06

**Authors:** William J Browne, Fiona Steele, Mousa Golalizadeh, Martin J Green

**Affiliations:** University of BristolUK; University of NottinghamUK

**Keywords:** Discrete time survival models, Event history models, Hierarchical centring, Markov chain Monte Carlo methods, Multilevel modelling, Orthogonal transformations

## Abstract

We consider the application of Markov chain Monte Carlo (MCMC) estimation methods to random-effects models and in particular the family of discrete time survival models. Survival models can be used in many situations in the medical and social sciences and we illustrate their use through two examples that differ in terms of both substantive area and data structure. A multilevel discrete time survival analysis involves expanding the data set so that the model can be cast as a standard multilevel binary response model. For such models it has been shown that MCMC methods have advantages in terms of reducing estimate bias. However, the data expansion results in very large data sets for which MCMC estimation is often slow and can produce chains that exhibit poor mixing. Any way of improving the mixing will result in both speeding up the methods and more confidence in the estimates that are produced. The MCMC methodological literature is full of alternative algorithms designed to improve mixing of chains and we describe three reparameterization techniques that are easy to implement in available software. We consider two examples of multilevel survival analysis: incidence of mastitis in dairy cattle and contraceptive use dynamics in Indonesia. For each application we show where the reparameterization techniques can be used and assess their performance.

## 1 Introduction

Survival analysis (which is also known as event history analysis) is widely used in the medical and social sciences to study the duration until the occurrence of events such as death, recovery from illness, unemployment, birth and divorce. The simplest form of survival model accounts for individuals who have not yet experienced the event by the end of the study period (right censoring) and time varying covariates, but survival data often have more complex features that researchers will wish to account for and to explore in their analyses. Events may occur several times to an individual over the study period, leading to *repeated events*, there may be *multiple states* between which individuals move (e.g. between employment and unemployment), an individual may be exposed to *competing risks* (e.g. different reasons for leaving a job) and there may be multiple *correlated processes* (e.g. the presence of children, outcomes of a birth history, may affect employment transitions and vice versa). Additional complexity will arise if durations are clustered in higher level units. Repeated events can be viewed as having a two-level hierarchical structure with episodes of exposure to the risk of an event nested within individuals, and individuals may themselves be nested within geographical areas or institutions. Random-effects or multilevel models are powerful tools for handling such features of survival data and have been proposed for the analysis of clustered durations (Clayton and Cuzick, 1985; Guo and Rodriguez, 1992; Sastry, 1997), competing risks and multiple states (Steele *et al.*, 1996, 2004) and the simultaneous analysis of correlated event processes (Lillard, 1993).

Although continuous time models, such as the Cox proportional hazards model (e.g. Hosmer and Lemeshow (1999), chapter 3), remain the most widely applied, discrete time approaches are increasingly used, especially in the social sciences. There are two main reasons for the popularity of discrete time methods. First, survival data are commonly collected retrospectively in cross-sectional surveys or prospectively in irregularly spaced waves of panel studies. Although the underlying event process is usually in continuous time, both forms of data collection lead to grouped or interval-censored event times that are most naturally analysed by using a model that recognizes the discrete nature of the data. Second, after some restructuring of the data, discrete time survival models are fitted by using standard methods for discrete response data, such as logistic regression. Consequently existing estimation procedures, implemented in mainstream and specialist statistical software packages, can be used to fit multilevel discrete time models for repeated events, multiple states, competing risks and correlated events. Applied researchers can now choose from a range of classical and Bayesian methods of estimation, including quasi-likelihood methods (e.g. Goldstein (1991)), adaptive quadrature (e.g. Skrondal and Rabe-Hesketh (2004)), *h*-likelihood (Lee and Nelder, 1996), simulated maximum likelihood (Ng *et al.*, 2006) and Markov chain Monte Carlo (MCMC) methods. In particular, the modular nature of MCMC algorithms make them an attractive choice for estimating models that account simultaneously for the types of complexity that were mentioned above. They have also been shown (Browne and Draper, 2006) to give less biased estimates than quasi-likelihood methods for random-effects logistic regression models.

Discrete time models are fitted to an expanded data file in which each duration is converted to a sequence of discrete responses, usually binary, indicating for each time interval whether an event has occurred. A drawback of the discrete time approach is that the restructured data file can be very large when the width of time intervals is short relative to the length of the observation period. As a result, estimation of multilevel discrete time models can be highly computationally intensive, especially when MCMC methods are used. MCMC methods produce correlated chains of parameter estimates and the length of chains that is required for accurate estimates is inversely related to this correlation. There are in fact many potential MCMC algorithms and one focus of methodological research has been the development of algorithms that reduce the correlation in the Markov chains for particular problems. Reducing this correlation results in having to run the chains for fewer iterations but this must be balanced by the (potential) increase in time per iteration due to the added complexity of the algorithm.

In this paper we investigate three methods to increase the computational efficiency of MCMC estimation of multilevel discrete time survival models: hierarchical centring (Gelfand *et al.*, 1995), orthogonal polynomials (e.g. Hills and Smith (1992)) and parameter expansion (Liu *et al.*, 1998). Although these approaches have all been described in the MCMC literature, they appear less frequently in applied journals and have not to our knowledge been applied specifically to multilevel survival models. Our aim is therefore to raise awareness among the survival modelling community of the potential of these methods and, through applications in veterinary medicine and demography, to illustrate their particular advantages (and disadvantages) in the analysis of two large and complex discrete time data sets.

The two applications have been chosen to illustrate a range of common features of survival data, and one aim of the paper is to investigate how the various approaches to improve MCMC efficiency perform for different data structures. In the first application, we use a three-level model to study between-year and between-farm variation in the incidence of mastitis in dairy cattle. The second application is to a study of discontinuation of contraception among Indonesian women. The data have a two-level structure with repeated episodes of contraceptive use nested within women, but the hierarchy is sparse with a high proportion of women contributing only one episode over the 6-year observation period. Although hierarchical centring leads to impressive gains in efficiency in the mastitis analysis, it performs poorly in the discontinuation of contraception example where the number of woman-specific random effects is large and the between-woman variance is small. This leads us to consider alternative strategies: orthogonal parameterizations and parameter expansion.

The remainder of the paper is structured as follows. In Section 2 we describe the discrete time approach to the analysis of clustered survival data. This is followed, in Sections 3 and 4, by the two applications of multilevel discrete time survival analysis. Hierarchical centring, orthogonal parameterizations and parameter expansion are described in the context of these applications, and the performance of each method is assessed and compared for the examples. We conclude in Section 5 with some general remarks and discussion.

## 2 Multilevel discrete time survival analysis

In both of the applications that we consider, the event of interest is repeatable: cows may suffer from mastitis on more than one occasion and women can initiate and discontinue use of contraceptives several times. In each case, the outcome is the duration until an event occurs, measured from the time that a cow or woman becomes at risk of experiencing the event. After an event occurs to an individual, a new episode begins if and when they subsequently become exposed to the risk of another event, leading to multiple episodes within individuals. In the mastitis application an episode is the duration until mastitis, whereas in the contraceptive use example an episode is defined as a continuous period of using the same method of contraception. We begin with a description of a two-level model for repeated events, although the same model can be applied to any two-level nested structure. In Section 3, we show how this model can be extended to accommodate a further hierarchical level. For further details of discrete time survival analysis see Allison (1982) and Singer and Willett (2003). The extension to random-effects models for the analysis of clustered data was discussed by Steele *et al.* (1996, 2004) and Barber *et al.* (2000).

Suppose that event times (i.e. lengths of episodes) are realizations of a random variable *T* measured in intervals of time indexed by *t*=1,…,*K* where *K* is the maximum duration of any episode. Denote by *t*_*ij*_ the number of intervals for which individual *j* is observed in episode *i*. Before carrying out a discrete time analysis, each episode *ij* must be expanded to obtain *t*_*ij*_ records. For each record, *t*=1,…,*t*_*ij*_, we define a binary variable *y*_*tij*_ which equals 1 if episode *ij* ends with an event during interval *t* and 0 otherwise. Thus all episodes will have *y*_*tij*_=0 for intervals *t*=1,…,*t*_*ij*_−1 and the response in the last observed interval *t*_*ij*_ will be 1 for episodes that end in an event and 0 for censored episodes. To give an example of the required data structure for our analysis of use of contraceptives where the event of interest is discontinuation, consider a woman who discontinues during the fourth month of use, then resumes use (after some time) and is still using the same method 3 months later at interview. This individual will have two episodes and, if monthly intervals are used, she will contribute seven records to the expanded discrete time file with response vector (0,0,0,1,0,0,0) for time intervals (1,2,3,4,1,2,3). (Note that the duration ‘clock’ restarts when she resumes use of contraception and starts a new episode.)

In a discrete time analysis, interest centres on the probability of event occurrence—the discrete time analogue of the continuous time hazard function. The discrete time hazard function is defined as 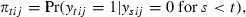 which is the conditional probability of an event in interval *t* given that no event has occurred in any previous interval of episode *ij*. In this paper we consider logit models for the dependence of *π*_*tij*_ on the duration of episode *ij* at the start of interval *t* and on covariates **x**_*tij*_, although extensions to other link functions are possible. A two-level random-effects logit model can be written: 
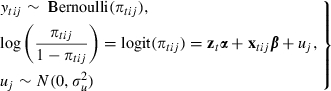
(1) where **z**_*t*_ is a vector of functions of the duration of the episode at *t* with associated coefficient vector ***α***, **x**_*tij*_ is a vector of covariates with coefficients ***β***, and *u*_*j*_ is an individual-specific random effect representing unobserved time invariant individual characteristics that affect the probability of an event throughout the observation period. Common choices for the baseline logit hazard **z**_*t*_***α*** include polynomials in *t* and step functions which result from treating *t* as a categorical variable. The covariates **x**_*tij*_ may be attributes of individuals, episodes or time intervals (i.e. time varying).

Allison (1982) showed that the likelihood function for a single-level discrete time model coincides with the binomial likelihood, and this equivalence generalizes to clustered data. Therefore a discrete time model can be fitted by using standard methods for clustered binary data, where the response variable for the analysis is the binary indicator of event occurrence *y*_*tij*_ in the expanded data set.

Model (1) is known as a proportional odds model because the effect of a time invariant covariate *x*_*kij*_ is assumed to be the same for each time interval *t*. The proportional odds assumption (which is analogous to proportional hazards in a model for the log-hazard) implies that, conditional on other covariates, the difference in the logit hazard of an event for two episodes with different values of *x*_*kij*_ does not depend on time. A non-proportional effect can be accommodated by including interactions between *x*_*kij*_ and elements of the duration vector **z**_*t*_. Other generalizations include random-coefficient models which allow the between-individual variance to depend on duration or covariates, and the addition of further levels (as in our application to mastitis in cows). The model can also be extended to handle competing risks, for example to distinguish between different reasons for discontinuation of contraception (Steele *et al.*, 1996), by defining *y*_*tij*_ as a categorical variable indicating event occurrence and type of event and fitting a multinomial logit model.

Although discrete time methods have various attractions, a potential disadvantage is the size of the expanded data set. Estimation of random-effects discrete time models can therefore be extremely slow. One strategy to reduce the size of the discretized file, and thus estimation times, is to aggregate intervals. Suppose, for example, that a woman discontinues contraception in the 10th month, leading to the following sequence of binary responses for the 10 months of exposure: (0,0,0,0,0,0,0,0,0,1). If we consider 6-monthly instead of monthly intervals, her 10 records will be collapsed to two—one for the first 6-month interval, and another for the second—and her new response vector will be (0,1). To allow for the fact that she was exposed to the risk of discontinuation for only 4 months of the second 6-month interval, we define an exposure vector which is coded (6,4). The aggregated binary response can then be analysed by using methods for binomial (grouped binary) data where exposure time within an interval is the denominator (Steele *et al.*, 2004). In aggregating intervals, however, we must assume that the hazard function and values of time varying covariates are constant within grouped intervals. In practice the second of these assumptions can have a sizable effect on the estimated effects of time varying covariates because of a loss of information on the relative timing of a change in the covariate value and an event.

In this paper, we consider an alternative approach which uses all available data. Using hierarchical centring, time invariant predictors can be centred on the individual level random effects which can both speed up the algorithm and in certain cases improve the mixing of the chains. We also describe two other methods that have the potential to improve the efficiency of the MCMC algorithm. The first is to orthogonalize the predictor variables, which in our experience has almost universal benefit at virtually no computational cost. The second is parameter expansion which in nested models appears to work best in exactly the cases that hierarchical centring does not, namely models with small between-cluster variability. We shall motivate orthogonalization in our first example and use both methods in our second example.

## 3 Application 1: incidence of mastitis in dairy cattle

Mastitis is an inflammation of the mammary gland of dairy cows, which is usually caused by a bacterial infection. Clinical cases of mastitis in early lactation often result from infections that arise during the previous non-lactating (dry) period and thus methods of farm management during the dry period are of interest in prevention of mastitis. Green *et al.* (2007) considered the use of multilevel survival models to investigate how cow, farm and management factors during the dry period influence the incidence of clinical mastitis after calving.

The data were collected over a 2-year period from 52 commercial dairy farms throughout England and Wales. For the analysis they distinguished cows that were housed during the dry period from those that were at pasture because many predictor variables were different for the two scenarios. Here we consider only housed cows, which results in a total of 8710 cow dry periods after which cases of mastitis were recorded from 103 farm-years in the 52 farms. (One farm had no housed cows in one year.) The data were expanded so that a discrete time survival model could be fitted with each interval being a week of lactation. This resulted in a total of 256382 records.

In Green *et al.* (2007) many predictor variables were considered. Each predictor was considered individually to establish whether there was any association with the response while accounting for the underlying nested structure. Predictors with a strong association were then considered together and a variable was retained in the model if its associated odds ratio had a 95% credible interval that did not include 1.0. After this process each discarded variable was reintroduced into the model to ensure that no effect was overlooked. Variation of the effect of predictors over farms and farm-years was assessed and this led to the inclusion of an additional random effect for parity 1 cows (cows that have given birth only once) at the farm level to explain additional variability in this subgroup when compared with the remainder of the animals. This makes biological sense as these cows only join the main herd after first calving and so less is known about their previous management. The alternative hierarchy of periods nested within cows nested within farms was also considered but this was discounted because of the worse model fit (according to the deviance information criterion diagnostic (Spiegelhalter *et al.*, 2002)) and the maximum of two periods per cow making identification of cow effects difficult. We consider the final model that was presented in Green *et al.* (2007) which has the form 
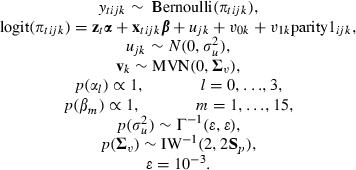


The hierarchical structure is as follows: weeks indexed by *t* nested within cow dry period *i*, nested within farm years *j*, nested within farms *k*. Here cow dry period plays the role of episode in Section 2 as it is assumed that a cow can have only one occurrence of mastitis per dry period. The binary response *y*_*tijk*_ takes value 1 if a case of clinical mastitis is observed in week *t* of cow dry period *i* and 0 otherwise. **z**_*t*_ consists of a constant plus polynomials in (centred) log-time to order 3 (to capture the effect of duration), **x**_*tijk*_ consists of 15 predictors that will be detailed later with associated effects ***β***. *u*_*jk*_ are the farm-year random effects, and *v*_0*k*_ are the farm random effects with different farm effects *v*_1*k*_ for the parity 1 cattle. The variance matrix of these sets of random effects has an inverse Wishart prior where **S**_*p*_ is an estimate of the farm level variance matrix obtained by using quasi-likelihood methods. The choice of a ‘default’ prior for a variance matrix is a rather open question (see Browne and Draper (2000), which contains our chosen prior as a possible choice) and we recommend testing the sensitivity of estimates to different prior specifications.

The predictors in the final model are as follows: parity of the cow (four dummy variables, parity1–parity4, to represent 1–4 previous births *versus* a base category of greater than 4); two dummy variables to indicate whether one or more somatic cell count readings were high before drying off the cows (scchigh) and whether at least two readings were available (scc>2); an indicator about whether farms ensure that cows remain standing for 30 min after administration of dry cow treatments (dostand); two dummy variables to indicate whether cubicle bedding is disinfected in the early dry period (edpdisinfect) and whether this is not applicable because of the system that was used (edpdisinfectna); two dummy variables to indicate whether transition cow cubicles are bedded at least once daily (transcow) and whether there are transition cow cubicles (transcowna); a dummy variable about whether the cubicle bedding is disinfected in the transition dry period (transdis); finally, three dummy variables to indicate whether the transition cubicle feed and loaf area is scraped daily (scrape1), more often than daily (scrape2) or does not exist (nofeedandloaf).

### 3.1. Hierarchical centring

Hierarchical centring (Gelfand *et al.*, 1995) is a type of reparameterization algorithm. The aim of such algorithms is to replace the original parameters in a model with new parameters that are less correlated with each other in the joint posterior distribution. An MCMC algorithm is then created for the new parameterization and Markov chains for the new parameters are produced. For the algorithm to work it must then be possible to transform these parameter chains to obtain chains for the original parameters.

Hierarchical centring uses the fact that multilevel models contain a linear predictor consisting of variables with associated fixed effects and zero-mean random effects. If a predictor is constant within clusters that are associated with the random effects then a simple reparameterization involves centring the random effects around it, i.e. replacing, in the linear predictor, the cluster level predictors and fixed effects and the zero-mean random effects with a new set of random effects; the mean of these new random effects is a function of the original cluster level predictors and fixed effects.

In our example we have a three-level model so there is potential for centring at either higher level (farm-year and farm). However, as the majority of predictors are defined at the farm-year level (management practice can change between years) it makes most sense to centre at this level. Of the predictors that were considered, all are defined at the farm-year level apart from the duration parameters, the parity indicators (parity1–parity4) and the somatic cell count predictors (scchigh and scc>2). The centred model is 
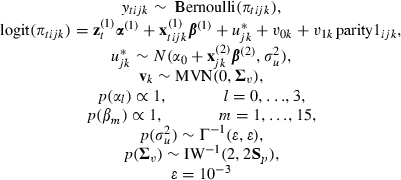
 where ***β***^(2)^ and ***β***^(1)^ are disjoint subsets of *β* representing the effects that can and cannot be centred, and ***α***^(1)^ contains all the duration effects apart from the constant, *α*_0_. For ease of comparison in the tables that follow we use the same ordering of the ***α***- and ***β***-vectors throughout.

To link between the two parameterizations in the centred model we have defined 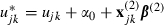 and so, whereas the non-centred algorithm will update *u*_*jk*_, the centred algorithm updates 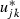. This model was fitted both in its non-centred and its centred forms by using MCMC estimation in the MLwiN software package (Rasbash *et al.*, 2000; Browne, 2003) and for the interested reader the algorithm for MCMC updating of a simpler hierarchically centred two-level model is given in Appendix A. Hierarchical centring also speeds up the algorithm because the time invariant predictors will be linked to the response indirectly via episode level effects. The update steps for these predictors hence only depend on these effects rather than the full response vector as is shown in Appendix A.

To compare the MCMC efficiency both here and in later applications we consider the effective sample size (ESS) diagnostic (Kass *et al.*, 1998), which is derived from the auto-correlation time *κ* with respect to the MCMC chain. This is defined as 
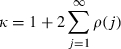
 where *ρ*(*j*) is the auto-correlation at lag *j*. We approximate this quantity by 
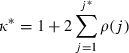
 where j^*^ corresponds to the first value *j*>5 such that *ρ*(*j*)<0.1. The ESS is then obtained by multiplying the actual number of iterations by κ^*^.

Table 1 gives the parameter estimates for 50000 iterations following a burn-in of 500 iterations, along with ESS values for each parameter.

**Table 1:** MLwiN results for the mastitis incidence survival model run for 50000 iterations†

*Parameter*	*Results for the non-centred model*		*Results for the hierarchically centred model*	
	*Estimate*‡	*ESS*	*Estimate*‡	*ESS*
*α*_0_—intercept	−2.650 (0.424)	*32*	−2.743 (0.400)	*2695*
*α*_1_—logt	−0.631 (0.033)	875	−0.632 (0.034)	926
*α*_2_—logt^2^	−0.462 (0.040)	98	−0.468 (0.043)	75
*α*_3_—logt^3^	−0.178 (0.013)	99	−0.181 (0.014)	75
*β*_1_—parity 1	−0.839 (0.124)	1095	−0.839 (0.126)	1058
*β*_2_—parity 2	−0.407 (0.055)	3747	−0.405 (0.054)	2087
*β*_3_—parity 3	−0.319 (0.055)	4759	−0.318 (0.055)	3275
*β*_4_—parity 4	−0.196 (0.056)	5165	−0.195 (0.056)	4271
*β*_5_—scchigh	0.329 (0.044)	2765	0.332 (0.044)	1438
*β*_6_—scc>2	0.030 (0.097)	1386	0.033 (0.097)	1248
*β*_7_—dostand	−0.356 (0.103)	*291*	−0.342 (0.102)	*3187*
*β*_8_—edpdisinfect	−0.279 (0.136)	*703*	−0.274 (0.145)	*3554*
*β*_9_—edpdisinfectna	0.163 (0.291)	*43*	0.155 (0.288)	*5359*
*β*_10_—transcow	−1.067 (0.326)	*44*	−1.022 (0.308)	*3448*
*β*_11_—transcowna	−1.382 (0.372)	*34*	−1.291 (0.362)	*2674*
*β*_12_—transdis	−0.650 (0.248)	*118*	−0.613 (0.249)	*2268*
*β*_13_—scrape1	−0.843 (0.292)	*57*	−0.793 (0.288)	*2524*
*β*_14_—scrape2	−0.885 (0.399)	*288*	−0.839 (0.393)	*1945*
*β*_15_—nofeedandloaf	−0.083 (0.296)	*50*	−0.101 (0.294)	*5183*
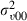	0.039 (0.019)	630	0.041 (0.020)	764
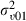	0.025 (0.024)	1555	0.026 (0.024)	1566
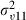	0.158 (0.065)	2439	0.154 (0.064)	2275
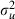	0.066 (0.022)	796	0.065 (0.022)	1052

† ESS values in italics are fixed effects associated with higher level predictors and hence are directly affected by centring.

‡ Standard deviations are given in parentheses.

In Table 1 the coefficients that are influenced by the centring have their ESS values italicized. It is obvious from a close inspection that the centring has increased all of these ESSs—many by a factor of over 100. In terms of computation time the non-centred model took over 19 h for 50000 iterations whereas the centred model took 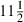 h—not quite halving the time. Note that 10 of the 19 fixed effects were affected by centring and each of the three sets of random effects takes a similar amount of computation as one fixed effect and so the time for a centred fixed effect is negligible compared with a non-centred fixed effect; this makes sense as in the MCMC updates for the centred fixed effects we only evaluate the farm-year level likelihood of 103 (normally distributed) farm-year level units whereas for the non-centred fixed effects we evaluate the likelihood for the 256382 (Bernoulli-distributed) level 1 units.

In general the reduction in the computation time that can be achieved through centring will clearly depend on both the number of fixed effects that can be centred and the ratio of level 1 to higher level units. This application does particularly well on both counts and so hierarchical centring makes real gains here.

Looking more closely at the ESS values in Table 1 we see that, among parameters that are unaffected by centring, those which are associated with duration have the lowest ESS. We shall now consider a method for improving the ESS values for these three parameters in this example. This will then motivate a more general use of orthogonalization of predictors that we use in our second example.

### 3.2. Orthogonal polynomials

In the above model we have included three terms to account for different risks as duration changes. These predictors, *z*_1*t*_=logtime_*t*_, 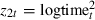 and 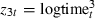, are highly correlated with pairwise correlations of −0.61, 0.79 and −0.90. We can, without altering the rest of the analysis, use a reparameterization that replaces this group of predictors with a less correlated group of predictors. In fact it makes sense to make the predictors orthogonal to each other, i.e.
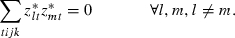


To do this we shall keep the first predictor 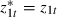 and then replace *z*_2*t*_ by 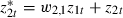 and 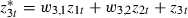 where the *w*-coefficients can be found uniquely so that the z^*^-predictors are orthogonal (see the second application for further details). If all predictors are centred then orthogonal predictors are also uncorrelated, but here, although **logtime** has been centred, **logtime^2^** and **logtime^3^** are not centred. In this example the orthogonal predictors are **logtime**, **logtime^2^**+0.94 **logtime** and **logtime^3^**+1.50 **logtime^2^**−2.05 **logtime**.

We can fit the full model as before but with the three z^*^-predictors replacing the **z**-predictors for duration. This will result in fixed effects 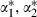 and 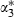 for these predictors but no change in the rest of the model. We can then transform back to the original parameters by creating chains for *α*_1_,*α*_2_ and *α*_3_ as follows: 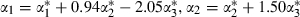 and 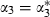.

Table 2 gives results from this method and, for comparison, the original parameterization. Here we see that the reparameterization improves the ESS for the duration parameters and now all parameters of the model have a more reasonable ESS.

**Table 2:** MLwiN results for the duration parameters in the mastitis incidence survival model after reparameterization and hierarchical centring†

*Parameter*	*Results for standard polynomials*		*Results for orthogonal polynomials*	
	*Estimate*‡	*ESS*	*Estimate*‡	*ESS*
*α*_0_—intercept	−2.743 (0.400)	*2695*	−2.711 (0.401)	*3337*
*α*_1_—logt	−0.632 (0.034)	926	−0.633 (0.033)	1997
*α*_2_—logt^2^	−0.468 (0.043)	75	−0.469 (0.039)	779
*α*_3_—logt^3^	−0.181 (0.014)	75	−0.181 (0.013)	1038

† The models were run for 50000 iterations.

‡ Standard deviations are given in parentheses.

## 4 Application 2: discontinuation of contraception in Indonesia

Steele *et al.* (2004) used multilevel multistate models to study transitions in and out of contraceptive use in Indonesia. In this paper, we consider a simplification of their model which considers only the transition from use to non-use: discontinuation of contraception. The data come from the 1997 Indonesia Demographic and Health Survey (Central Bureau of Statistics *et al.*, 1998) which is a representative survey of all married women between the ages of 15 and 49 years. Contraceptive use histories were collected retrospectively for the 6-year period before the survey and include information on the month and year of starting and stopping use, the method used and the reason for discontinuation. The analysis is based on 17833 episodes of contraceptive use for 12 594 women, where an episode is defined as a continuous period of using the same method of contraception. Restructuring the data to discrete time format with monthly time intervals leads to 365205 records. To reduce the size of the data set, durations were grouped into 6-month intervals and analysed by using methods for binomial (grouped binary) data with denominator for grouped interval *t* equal to the number of months for which a woman was at risk of discontinuation (i.e. using contraceptives) during *t* (see Section 2 for an example). Aggregation of intervals leads to a data set with 68515 records.

If we let *y*_*tij*_ equal 1 if episode *i* of woman *j* ends in discontinuation during interval *t* and 0 otherwise and *n*_*tij*_ be the number of months for which woman *j* was at risk of discontinuation during interval *t* of episode *i*, a multilevel model for the associated probability of discontinuation *π*_*tij*_ (the hazard of discontinuation) that accounts for correlation between the durations of episodes that are contributed by the same woman can be written 
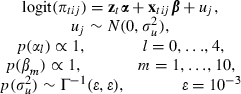
 where **z**_*t*_***α*** is a function of the duration of use at *t*, **x**_*tij*_ is a vector of covariates with corresponding fixed effects ***β***, and *u*_*j*_ are woman-specific random effects. Steele *et al.* (2004) found that a piecewise constant hazard was a good fit to the observed hazard, with five duration intervals of 0–5, 6–11, 12–23, 24–35 and 36 months or longer. Thus the baseline logit hazard **z**_*t*_***α*** is a step function, where **z**_*t*_ contains a constant and dummy variables for intervals 6–11, 12–23, 24–35 and 36 months or longer. The predictors **x**_*tij*_ include the woman's age at the start of the episode (less than 25, 25–34 and 35–49 years), contraceptive method (classified as pill or injectable, Norplant or intrauterine device, other modern and traditional), education (three groups), type of region of residence (urban or rural) and socio-economic status (coded as low, medium or high). These categorical predictors are represented by 10 dummy variables. Duration is time varying whereas age and method are episode level variables. All other variables (including the constant) are defined at the woman level and are therefore candidates for hierarchical centring, affecting six of the 15 coefficients in the model (the intercept, one for type of region, two for socio-economic status and two for education). As with the mastitis example we consider both a centred and a non-centred formulation of the model.

Table 3 shows the results for the non-centred and hierarchically centred formulations, based on 250000 iterations with every 10th iteration stored to reduce storage requirements.

**Table 3:** MLwiN results for the random-intercept model of discontinuation of contraception in Indonesia, 250000 iterations

*Parameter*	*Results for the non-centred model*		*Results for the hierarchically centred model*	
	*Estimate*†	*ESS*	*Estimate*†	*ESS*
*α*_0_—constant	−4.053 (0.078)	*1665*	−4.045 (0.091)	*28*
*α*_1_—duration6–11	−0.067 (0.045)	13405	−0.069 (0.045)	91
*α*_2_—duration12–23	−0.117 (0.043)	11553	−0.121 (0.042)	81
*α*_3_—duration24–35	−0.022 (0.051)	11900	−0.030 (0.050)	126
*α*_4_—duration≥36	0.043 (0.057)	10463	0.031 (0.055)	161
*β*_1_—age25–34	−0.375 (0.033)	13855	−0.373 (0.033)	163
*β*_2_—age35–49	−0.676 (0.058)	15933	−0.673 (0.058)	11840
*β*_3_—method2	−1.164 (0.059)	19911	−1.159 (0.059)	20562
*β*_4_—method3	0.499 (0.110)	20517	0.496 (0.109)	18820
*β*_5_—method4	0.051 (0.062)	21118	0.051 (0.062)	19313
*β*_6_—educ–primary	0.028 (0.069)	*2036*	0.028 (0.071)	*50*
*β*_7_—educ–secondary+	0.223 (0.072)	*2093*	0.222 (0.073)	*44*
*β*_8_—urban	0.114 (0.037)	*16965*	0.117 (0.035)	*79*
*β*_9_—ses–med	−0.118 (0.046)	*6488*	−0.119 (0.052)	*33*
*β*_10_—ses–high	−0.192 (0.052)	*6876*	−0.188 (0.053)	*55*
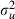	0.041 (0.026)	14	0.022 (0.018)	14

† Standard deviations are given in parentheses.

In this example using hierarchical centring actually has a detrimental effect with far worse ESS, not only for the predictors that are involved in the centring but also some of the other predictors. This application differs from the mastitis example in that we have far more random effects (women) than we had previously (farm-years) and these random effects have a very small variance. It is striking that the worst ESS is for the woman-level variance parameter. It is well known that hierarchical centring does not work well when the random-effect variance is small and Gelfand *et al.* (1995) showed this empirically for normal responses. An intuitive explanation for this is that with small (uncentred) random effects there will be strong correlations between the predictors representing the mean of the (centred) random effects and the centred random effects themselves and this will induce poor mixing of the chains.

Further examination of the data reveals a large number of right-censored observations, i.e. women who used contraceptives throughout the 6-year observation period, and a small number of women who start and stop using contraceptives very quickly on several occasions. This might call into question the normal distribution assumption for the woman effects. Here we shall first consider simply removing the random effects, as their variance is small, and examine the effect that this has on the fixed effect coefficients. Second, we shall investigate how we might improve mixing for the random-effect variance parameter under a normal distribution assumption.

### 4.1. Orthogonal parameterization

We begin by removing the random effects from the model and fitting a simple logistic regression model. If we remove the random effects it is impossible to use hierarchical centring as we have no hierarchical structure. We shall instead consider an extension of the orthogonal polynomial approach that was used in the analysis of the mastitis data set. In the mastitis model the ESS values for the duration fixed effects were improved by a reparameterization using orthogonal polynomials. Here we shall consider using the same approach on all the predictor variables. Note that orthogonal parameterizations have been used previously in Hills and Smith (1992) where the focus was on creating orthogonal parameters rather than orthogonal predictors.

In Appendix B we give details of an algorithm that takes all the fixed effect predictors in our model and transforms them into an orthogonal set of predictors, resulting in a reparameterized model. In brief the algorithm takes the set of all predictors **P** (constructed so that we can write **z**_*t*_***α***+**x**_*tij*_***β*** as **p**_*tij*_***θ*** where ***θ***=(***α***,***β***)) and creates a new set of orthogonal predictors P^*^. As the set of orthogonal predictors spans the same space as the original predictors we can easily then recover the fixed effects from the original parameterization. There is not one unique set of orthogonal predictors that can be generated in this way and in the algorithm the order that the predictors appear in **P** governs which set of predictors is generated in P^*^.

Table 4 gives results and the ESS for the simple logistic regression model fitted by using both the original predictors and an orthogonal set of predictors (with the estimates converted back to coefficients of the original predictors).

**Table 4:** MLwiN results for the simple logistic regression model for the Indonesia data set run for 50000 iterations

*Parameter*	*Results for original predictors*		*Results for orthogonal predictors*	
	*Estimate*†	*ESS*	*Estimate*†	*ESS*
*α*_0_—constant	−4.025 (0.073)	403	−4.037 (0.077)	11578
*α*_1_—duration6–11	−0.073 (0.045)	4249	−0.071 (0.045)	12049
*α*_2_—duration12–23	−0.128 (0.043)	3617	−0.126 (0.042)	11878
*α*_3_—duration24–35	−0.040 (0.050)	4643	−0.038 (0.050)	11920
*α*_4_—duration≥36	0.017 (0.055)	5260	0.017 (0.055)	11864
*β*_1_—age25–34	−0.371 (0.033)	5114	−0.371 (0.033)	12908
*β*_2_—age35–49	−0.672 (0.057)	7787	−0.670 (0.058)	10188
*β*_3_—method2	−1.115 (0.058)	10291	−1.156 (0.059)	8518
*β*_4_—method3	0.495 (0.109)	11306	0.492 (0.109)	12010
*β*_5_—method4	0.052 (0.062)	9877	0.052 (0.062)	12023
*β*_6_—educ–primary	0.021 (0.065)	500	0.030 (0.069)	10945
*β*_7_—educ–secondary+	0.215 (0.067)	514	0.225 (0.071)	11610
*β*_8_—urban	0.114 (0.036)	5646	0.113 (0.036)	10591
*β*_9_—ses–med	−0.119 (0.045)	1466	−0.116 (0.046)	11214
*β*_10_—ses–high	−0.193 (0.051)	1686	−0.191 (0.052)	10249

† Standard deviations are given in parentheses.

We now see an improvement in ESS for nearly all the predictors, with an ESS of around 10000–12000 for each. In fact it seems that using orthogonal predictors makes mixing (and hence the ESS) roughly the same for each parameter. For this model we ordered the predictors as follows: duration6–11, duration12–23, duration24–35, duration≥36, age25–34, age35–49, method2,method3, method4, constant, educ–primary, educ–secondary+, urban, ses–med, ses–high.

This ordering was chosen so that the level 1 predictors were picked first. It would be interesting in future research to investigate whether there is any way to choose a ‘best’ ordering for this algorithm (if indeed the ordering matters for this model). Our algorithm is a straightforward way of producing one set of orthogonal predictors, but there are many other ways of producing such predictors and again it would be interesting to investigate whether we can find an algorithm for finding the ‘best’ set of orthogonal predictors spanning the same space as the original predictors. In both cases we mean ‘best’ in the sense of producing the least auto-correlated chains.

Owing to the small between-woman variance, the removal of the random effects has had little effect on the fixed effects in the model (see Tables 3 and 4). The only fixed effects that have changed noticeably are those for the later duration categories although they are not statistically significant in either model. A possible explanation for this difference between the results of the random-effects and simple logistic models is unobserved heterogeneity between women, although we know from the random-effects analysis that the between-woman variance is small. Over time, the risk population will increasingly consist of women with a low risk of discontinuation, and it is these women who contribute to the estimation of the coefficients of the baseline hazard at longer durations. Failure to adjust for unobserved heterogeneity will lead to overstatement of negative duration effects and understatement of positive duration effects (Vaupel *et al.*, 1979).

If we return to the original model with woman level random effects we can explore whether we can improve MCMC efficiency by using orthogonal predictors. Because of its earlier poor performance in this application, we do not use hierarchical centring, and to create the orthogonal predictors we again use the ordering (as for the single-level model) which includes the level 1 predictors first followed by the woman level predictors. Thus by using the algorithm in Appendix B all the orthogonal predictors that are produced will now be at level 1. In Table 5 we give results of fitting this model in MLwiN.

**Table 5:** Results from MLwiN for the random-effects logistic regression model using orthogonal predictors and 250000 iterations thinned by factor 10

*Parameter*	*Estimate*†	*ESS*
*α*_0_—constant	−4.040 (0.078)	22714
*α*_1_—duration6–11	−0.071 (0.045)	23931
*α*_2_—duration12–23	−0.124 (0.043)	24136
*α*_3_—duration24–35	−0.035 (0.050)	23303
*α*_4_—duration≥36	0.022 (0.055)	22457
*β*_1_—age25–34	−0.371 (0.033)	23347
*β*_2_—age35–49	−0.671 (0.059)	22779
*β*_3_—method2	−1.156 (0.058)	22105
*β*_4_—method3	0.495 (0.108)	23533
*β*_5_—method4	0.052 (0.062)	24498
*β*_6_—educ–primary	0.029 (0.069)	23816
*β*_7_—educ–secondary+	0.224 (0.072)	23428
*β*_8_—urban	0.114 (0.036)	22860
*β*_9_—ses–med	−0.117 (0.046)	23697
*β*_10_—ses–high	−0.191 (0.052)	23624
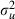	0.008 (0.006)	20

† Standard deviations are given in parentheses.

Once again we find that the use of orthogonal predictors has resulted in similar sized ESS for all fixed effects. This model is more complex than the simple logistic regression model without random effects and so the choice of ordering in our algorithm will be more important. This is because when choosing orthogonal predictors we would ideally like these also to be close to orthogonal to the woman identifiers in the model (0–1 vectors defining which observations belong to particular women). For example if one of the orthogonalized predictors was highly correlated with a woman identifier then in the likelihood the fixed effect and woman residual associated respectively with the pair would play similar roles. They would hence be highly correlated and this would mean that single-site updating of the parameters will result in poorly mixing chains. As observed previously in Table 3 the worst mixing parameter for this model is 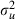. In the next section we discuss a method that might improve mixing for this variance parameter.

### 4.2. Parameter expansion

Parameter expansion was originally developed by Liu *et al.* (1998) to speed up the EM algorithm. This method was then considered in relation to MCMC sampling and the Gibbs sampler by Liu and Wu (1999) and has since been considered for random-effects models by Van Dyk and Meng (2001), Browne (2004) and Gelman *et al.* (2008). The basic idea of the technique is to embed the desired model of interest in a larger model by adding additional redundant parameters to the model. These parameters make the larger model unidentified but the embedded model is still identified and its parameters can be extracted. In the case of random-effects models parameter expansion is effective when the variance of the random effects has large mass near zero. In these problems there is strong correlation between the random-effects chains and the chain for their variance, and parameter expansion introduces an additional parameter that effectively updates both the random effects and their variance together.

A model with orthogonalization (as described in Appendix B) and parameter expansion is then 
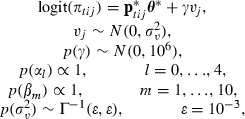
 and we can return to our original parameters by taking *u*_*j*_=*γv*_*j*_, ***θ***=(***α***,***β***)=**W**^T^θ^*^ and 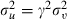 where **W** is defined in the algorithm in Appendix B.

For this section we switch software to WinBUGS (Spiegelhalter *et al.*, 2003) as parameter expansion is not currently implemented in MLwiN. In WinBUGS we select the implementation of the multivariate Metropolis–Hastings approach of Gamerman (1997) which is computationally slow but gives better mixing chains. As this method itself effects mixing we also fit in WinBUGS the parameterization without parameter expansion for comparison both with the MLwiN results in Section 4.1 and the parameter-expanded model. The MCMC estimates for both models are based on 25000 iterations following a burn-in of 500. The results are shown in Table 6 with the results without parameter extension in the left-hand columns and with parameter expansion in the right-hand columns.

**Table 6:** Results from WinBUGS for the random-effects logistic regression model using orthogonal predictors and parameter expansion and 25000 iterations

*Parameter*	*Results for the orthogonal only model*		*Results for the orthogonal and parameter expanded model*	
	*Estimate*†	*ESS*	*Estimate*†	*ESS*
*α*_0_—constant	−4.037 (0.077)	24537	−4.064 (0.081)	14009
*α*_1_—duration6–11	−0.071 (0.045)	25936	−0.065 (0.045)	22017
*α*_2_—duration12–23	−0.126 (0.042)	25251	−0.112 (0.044)	15024
*α*_3_—duration24–35	−0.038 (0.050)	25083	−0.014 (0.054)	4859
*α*_4_—duration≥36	0.018 (0.054)	26332	0.055 (0.063)	1881
*β*_1_—age25–34	−0.371 (0.033)	26567	−0.376 (0.034)	22609
*β*_2_—age35–49	−0.671 (0.058)	22787	−0.677 (0.059)	20883
*β*_3_—method2	−1.156 (0.059)	18532	−1.169 (0.060)	14032
*β*_4_—method3	0.494 (0.109)	24267	0.501 (0.111)	23488
*β*_5_—method4	0.053 (0.062)	24546	0.050 (0.063)	23792
*β*_6_—educ–primary	0.030 (0.069)	24012	0.029 (0.069)	22546
*β*_7_—educ–secondary+	0.224 (0.071)	25563	0.225 (0.072)	24422
*β*_8_—urban	0.113 (0.036)	25157	0.115 (0.037)	22995
*β*_9_—ses–med	−0.116 (0.046)	25302	−0.118 (0.046)	23960
*β*_10_—ses–high	−0.190 (0.052)	25139	−0.192 (0.052)	23383
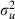	0.001 (0.001)	13	0.059 (0.048)	318

† Standard deviations are given in parentheses.

Comparing the results for MLwiN and WinBUGS by using the formulation without parameter expansion (Table 5 and Table 6, left-hand column) we see similar estimates and similar ESS values. As some chains have small negative auto-correlation, we even have some ESSs that are bigger than 25000. We ran MLwiN for 10 times as many iterations but because of the better mixing from the Gamerman method the ESS is approximately the same. However, MLwiN took 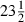 h whereas WinBUGS took 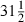 h and so the choice between methods is not clear cut. As we might expect, the worst mixing parameter is still 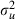 and switching software package has not fixed this.

**Fig. 1:**
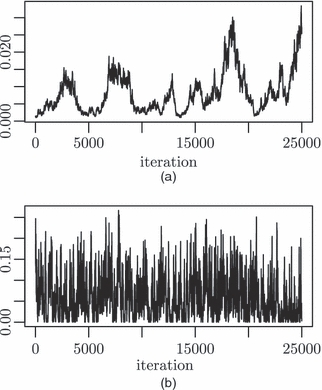
MCMC chains for the between-woman variance (a) before and (b) after parameter expansion

If we next compare the effect of the parameter expansion by comparing the two sets of results in Table 6 we finally see an improvement for 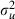. This is illustrated further by the MCMC chains for 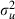 that can be seen in Fig. 1. The estimate of the variance has increased owing to the change of prior that occurs when parameter expansion is used. The ESSs for the fixed effects are all worse than those in the left-hand columns before parameter expansion and this makes sense as the random effects are larger and so correlations between these and the fixed effects will have more effect on the mixing of the chains. Parameter expansion is computationally more expensive, taking around 34 h for the 25000 iterations.

## 5 Discussion

In this paper we have examined fitting multilevel discrete time survival models to two large data sets from veterinary epidemiology and demography. We have seen promising improvements in both the speed and the efficiency of MCMC algorithms when an alternative hierarchically centred parameterization is used in the mastitis example. However, as is well known, hierarchical centring performs worse in cases when the cluster level variance is small, as is the case in the contraceptive use example. We find that transforming the fixed predictors to be orthogonal has a beneficial effect and the technique of parameter expansion also helps in this example.

The main differences between the two data sets are the numbers of level 2 units and the size of the level 2 variance. In the mastitis example we have only 103 farm-years for 256582 observations with significant variation at both the farm-year and the farm levels. For the contraceptive use data set we have 12594 women with 68515 6-monthly observations, but around two-thirds of the women (8701) never discontinued use during the study period. This means that we have very little information on individual women and hence problems in estimating the between-woman variance, which is estimated as very small. In this case there appears to be little gained from fitting random woman effects and a simple logistic regression model gives similar fixed effect estimates.

In the mastitis example we could in theory have fitted individual cow random effects (crossed with the farm-year effects as some cows appear in both years of the study) although this was ruled out on grounds of model fit in Green *et al.* (2007). We would have experienced similar problems with estimating the between-cow variance, and so this situation is not restricted to our contraceptive use example.

What we should emphasize here is that, for all fitted models, the methods that we have used have produced similar estimates. Our aim has been to obtain accurate estimates in fewer iterations, rather than to correct biased estimates. The technique of hierarchical centring, in our experience, works well in all cases apart from when we have very small higher level variance, supporting the empirical results in Gelfand *et al.* (1995) for normal response models, and has additional improvements in speed of execution.

Much research has followed on from Gelfand *et al.* (1995). For example Papaspiliopoulos *et al.* (2003, 2007) discussed partially non-centred parameterizations that can (for Gaussian response models) be shown to improve on both the centred and the non-centred parameterizations that we consider. They also suggested a way of constructing such a parameterization in the non-Gaussian context which would be worth considering in further work.

The method of transforming predictor variables so that they are orthogonal seems also to be a good reparameterization technique, at least in the examples that were considered here. More research is required on choosing ‘best’ sets of orthogonal predictors, although choices that are also close to orthogonal to the dummy variables representing the level 2 unit identifiers would seem preferable. It would also be feasible to combine the approach with hierarchical centring by producing two sets of orthogonal predictors from both the level 1 and the level 2 predictors.

In this paper we have focused on the application of reparameterization methods to the estimation of multilevel discrete time survival models, but the three methods that were considered should have similar effects on all forms of generalized linear mixed model. The random effects need not simply be nested, as Browne (2004) demonstrated the use of both hierarchical centring and parameter expansion in crossed random-effects models.

Although parameter expansion has clear benefits for models with small random-effects variance, it is debatable whether in such cases random effects are necessary. The concept of embedding an identified model within an unidentified model will probably seem a little difficult to some readers. However, an interesting alternative in the parameter expansion framework is to constrain the variance 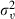 to be 1; the identifiability issue then disappears and *γ* now plays the role of the standard deviation of the random effects. This formulation results in a uniform prior for the standard deviation of the random effects which has recently become popular and has the same mixing benefits as parameter expansion.

## Appendix A: Markov chain Monte Carlo algorithm for a general two-level discrete time survival variance components logistic regression model

Let us assume that like the Indonesia example in Section 4 we have an initial two-level data set (events nested within women) and that we expand the data to give a three-level structure of time intervals nested within events nested within clusters (women). Then let us assume that the only random effects are at the cluster level and are simply changes in intercept and hence we have a variance components model. As no random terms are associated with events in reality we still have a two-level model (time intervals within clusters), and we shall assume that we fit a random-effects logistic regression model with *p*_2_ predictors at the cluster level (including the intercept) and *p*_1_ predictors at either the event level or time varying within events (including duration parameters) which for simplicity we shall group together. We can write such a model as

Here  contains the *p*_1_ predictors for occasion *t* in event *i* in cluster *j* and  contains the *p*_2_ cluster level predictors for cluster *j*.

Then to convert to a hierarchical centred formulation we have

where .

An MCMC sampling algorithm using this formulation but described in terms of the original parameters is as follows.

*Step 1*: update ***β***^(1)^ by using a univariate random-walk Metropolis step at iteration *s*+1 as follows. For *k*=1,…,*p*_1_ and with  signifying the ***β***^(1)^-vector without component *k*,

where ,  is the proposal variance for  which can be fixed or adapted by using an algorithm that was described in Browne and Draper (2000) and

*Step 2*: update the random effects *u*_*j*_ by using a univariate random-walk Metropolis step at iteration *s*+1 as follows. For *j*=1,…,*J* and with **u**_(−*j*)_ signifying the **u**-vector without component *j*,

where ,  is the proposal variance for *u*_*j*_ and

*Step 3*: update the random-effects variance  at iteration *s*+1 by drawing from the gamma full conditional distribution for ,

*Step 4*: update ***β***^(2)^ as a vector using Gibbs sampling at iteration *s*+1 from its full conditional distribution, which is multivariate normal with dimension *p*_2_,

where

and

and let

to ensure that  remains fixed. Here ***β***^(2)^(*s*) is the value of ***β***^(2)^ before the step and  is the value of *u*_*j*_ before updating ***β***^(2)^ (but after updating *u*_*j*_ in step 2).

## Appendix B: Algorithm for generating orthogonal predictors

For simplicity of exposition in the algorithm that follows we shall combine the duration effects and the other predictors into one matrix that we shall call **P** so that we can write **z**_*t*_***α***+**x**_*tij*_***β*** as **p**_*tij*_***θ*** where ***θ***=(***α***,***β***).

The algorithm that we use to produce orthogonal predictors follows on from the method for orthogonal polynomials and is as follows.

*Step 1*: number the predictors in some ordering 1,…,*N*.

*Step 2*: take each predictor in turn and replace it with a predictor that is orthogonal to all the (orthogonal) predictors already considered as described below.

For predictor **p**_*k*_ (the *k*th column of **P**) create  so that . This results in solving *k*−1 equations in *k*−1 unknown *w*-coefficients.

By performing this step for all predictors in turn we shall end up with  coefficients. Once we have performed step 2 for all predictors we have a lower diagonal matrix **W**=(*w*_*i*,*j*_) such that **P**^*^=**WP** and so we can run the model using the **P**^*^-predictors.

This will result in chains for parameters ***θ***^*^=(***α***^*^,***β***^*^) which can be transformed into chains for the original parameters ***α*** and ***β*** by premultiplying ***θ***^*^ by **W**^T^. Each unique ordering of the predictors will give a unique set of orthogonal predictors. The algorithm will generally work provided that the predictors are not collinear.

From a Bayesian modelling point of view it is worth pointing out here that we would normally need to calculate the Jacobian of the transformation from ***α*** and ***β*** to ***α***^*^ and ***β***^*^ to ensure that we maintain the same prior distributions after reparameterization. In our example, however, we use improper uniform priors and so this is not a problem.
